# Characterization and Process Optimization for Enhanced Production of Polyhydroxybutyrate (PHB)-Based Biodegradable Polymer from *Bacillus flexus* Isolated from Municipal Solid Waste Landfill Site

**DOI:** 10.3390/polym15061407

**Published:** 2023-03-12

**Authors:** Mohd Adnan, Arif Jamal Siddiqui, Syed Amir Ashraf, Mejdi Snoussi, Riadh Badraoui, Angum M. M. Ibrahim, Mousa Alreshidi, Manojkumar Sachidanandan, Mitesh Patel

**Affiliations:** 1 Department of Biology, College of Science, University of Ha’il, Ha’il P.O. Box 2440, Saudi Arabia; drmohdadnan@gmail.com (M.A.);; 2 Department of Clinical Nutrition, College of Applied Medical Sciences, University of Ha’il, Ha’il P.O. Box 2440, Saudi Arabia; 3 Department of Pharmaceutical Chemistry and Pharmacognosy, College of Pharmacy, Jazan University, P.O. Box 114, Jazan 45142, Saudi Arabia; 4 Department of Oral Radiology, College of Dentistry, University of Ha’il, Ha’il PO Box 2440, Saudi Arabia; 5 Department of Biotechnology, Parul Institute of Applied Sciences and Centre of Research for Development, Parul University, Vadodara 391760, India

**Keywords:** *Bacillus flexus*, biodegradable polymer, bioplastic, Box–Behnken, Ha’il, Polyhyroxybutyrate, response surface methodology

## Abstract

In recent years, there has been a growing interest in bio-based degradable plastics as an alternative to synthetic plastic. Polyhyroxybutyrate (PHB) is a macromolecule produced by bacteria as a part of their metabolism. Bacteria accumulate them as reserve materials when growing under different stress conditions. PHBs can be selected as alternatives for the production of biodegradable plastics because of their fast degradation properties when exposed to natural environmental conditions. Hence, the present study was undertaken in order to isolate the potential PHB-producing bacteria isolated from the municipal solid waste landfill site soil samples collected from the Ha’il region of Saudi Arabia to assess the production of PHB using agro-residues as a carbon source and to evaluate the growth of PHB production. In order to screen the isolates for producing PHB, a dye-based procedure was initially employed. Based on the 16S rRNA analysis of the isolates, *Bacillus flexus* (*B. flexus*) accumulated the highest amount of PHB of all the isolates. By using a UV–Vis spectrophotometer and Fourier-transform infrared spectrophotometer (FT-IR), in which a sharp absorption band at 1721.93 cm^−1^ (C=O stretching of ester), 1273.23 cm^−1^ (–CH group), multiple bands between 1000 and 1300 cm^−1^ (stretching of the C–O bond), 2939.53 cm^−1^ (–CH_3_ stretching), 2880.39 cm^−1^ (–CH_2_ stretching) and 3510.02 cm^−1^ (terminal –OH group), the extracted polymer was characterized and confirmed its structure as PHB. The highest PHB production by *B. flexus* was obtained after 48 h of incubation (3.9 g/L) at pH 7.0 (3.7 g/L), 35 °C (3.5 g/L) with glucose (4.1 g/L) and peptone (3.4 g/L) as carbon and nitrogen sources, respectively. As a result of the use of various cheap agricultural wastes, such as rice bran, barley bran, wheat bran, orange peel and banana peel as carbon sources, the strain was found to be capable of accumulating PHB. Using response surface methodology (RSM) for optimization of PHB synthesis using a Box–Behnken design (BBD) proved to be highly effective in increasing the polymer yield of the synthesis. With the optimum conditions obtained from RSM, PHB content can be increased by approximately 1.3-fold when compared to an unoptimized medium, resulting in a significant reduction in production costs. Thus, isolate *B. flexus* is a highly promising candidate for the production of industrial-size quantities of PHB from agricultural wastes and is capable of removing the environmental concerns associated with synthetic plastics from the industrial production process. Moreover, the successful production of bioplastics using a microbial culture provides a promising avenue for the large-scale production of biodegradable and renewable plastics with potential applications in various industries, including packaging, agriculture and medicine.

## 1. Introduction

Pollution caused by plastic has grown into one of the biggest concerns on a global scale due to the considerable impact it has on the environment. Plastics made from petrochemicals are affordable, durable materials that are widely used for a variety of purposes, from packaging to aerospace [1,2,3,4]. In spite of this, most plastics (including polycarbonate, polyvinyl chloride, polystyrene and polyethylene) cannot degrade in the short term or even over a long period of time because their long stable polymer chains make them very difficult to degrade [5]. In consequence, it is estimated that by 2025 there will be 11 billion metric tons of plastics accumulating in landfills and in the natural environment, causing severe pollutants to enter the natural environment. As one of the largest markets for plastics, packaging can be defined as an application whose growth has been accelerated by increasingly single-use containers being used instead of reusable containers [6]. Due to the growing use of plastic materials, the proportion of plastics in municipal solid wastes has increased from less than 1% in 1960 to more than 20% by 2022 in countries with middle and high incomes [7]. Furthermore, over the past five decades, the production of solid waste has also grown steadily [8,9].

A large proportion of the monomers used in the manufacture of plastics, such as ethylene and propylene, are derived from fossil hydrocarbons. All common plastics are non-biodegradable. Consequently, landfills or the natural environment become overburdened with garbage and refuse rather than decomposing with time [10]. Plastic waste can be permanently eliminated through destructive thermal treatment, such as combustion or pyrolysis. It is, therefore, a growing concern that plastic waste is going to pollute the natural environment in a near-permanent way. Moreover, there have been reports of plastic debris found in every major ocean basin [11]. It is also becoming increasingly apparent that freshwater systems and terrestrial habitats are also at risk of contamination [12,13,14], as is the presence of synthetic fibers in the environment [14,15]. As a result of the abundance of plastic waste in the environment today, it has been proposed that the presence of plastic waste is a geological indicator of the proposed Anthropocene era [16].

Consequently, as a replacement for synthetic plastics, bioplastics are now in heavy demand because they are non-toxic, renewable, biocompatible, and biodegradable, thereby gaining popularity in recent years [17]. There are various renewable raw materials that can be used to develop biobased plastics, including polysaccharides and proteins, plants (starch-based plastics and cellulose-based plastics) and microbial bioplastics (polylactic acid and polyhydroxyalkanoates (PHAs)) [18]. There are several forms of PHAs, but the most common is poly-3-hydroxybutyrate (PHB), which is an organic compound that is synthesized by microbes by binding β-hydroxybutyrate monomers to ester bonds [19]. PHB is used in many different technologies as an interior component for electrical appliances, sanitary goods, beverage containers, packaging, coating materials, and for disposable substances [20].

Nevertheless, there have been recent advances in the field of medicine involving the design and biosynthesis of antimicrobial materials containing PHB nanocomposites and silver nanoparticles [21] and used as drug carriers for wound management and tissue engineering [22]. It is generally recognized that PHB materials are stiff and brittle in nature, as well as exhibiting a high degree of crystallinity and low thermal stability. The properties of many PHB plastics are very similar to those of the petroleum polymers polypropylene (PP) and polyethylene (PE). PHB biopolymers can be produced with feedstocks that are renewable and sustainable, such as food waste. A combination of these factors, together with PHB’s ability to be biocompatible and its tendency to biodegrade when exposed to certain active biological environments, make it an excellent alternative to synthetic polymers like PVC, PP, and PE [23]. 

However, PHBs have a high production cost relative to plastics derived from petrochemicals, which prevents their extensive production and commercialization. It has been recently discovered that there are several ways to reduce the production cost of PHB, including the development of efficient bacterial strains, the optimization of fermentation and the recovery process [24,25]. Most reports on PHB production suggest that carbon substrate costs account for the majority of PHB production costs [26]. It is, therefore, crucial to select carbon substrates that are efficient, as this determines the total production cost. Alternatively, renewable carbon substrates are available, economical and most readily available for both microbial growth and effective PHB synthesis [24,26]. Accordingly, the objective of this study was to isolate bacteria that produce PHB from agricultural waste materials and to study their production of PHB.

A number of previous studies have been conducted on the isolation of PHB and its production through the use of agricultural residues. The PHB-producing *Bacillus* sp. isolated by Getachew and Woldesenbet [27] was capable of producing PHB from banana peels, corn cobs, sugarcane bagasse, corn cobs and Teff (*Eragrostis teff*) straws. *B. cereus* was isolated from the Kafr El-sheikh Governorate, Egypt, by Belal and Farid [28], which produces PHBs when inoculated in a medium supplemented with 2% glucose, xylose, lactose, whey, molasses, sugar cane bagasse, and rice straw. The marine bacterium *Pseudodonghicola xiamenensis* has been isolated by Mostafa et al. [29], which is capable of producing PHB from Date syrup at a low cost. In another study, Mostafa et al. [30] reported novel strains of PHB-producing bacteria isolated from the mangrove rhizosphere in the Red Sea, Saudi Arabia. These strains were *Tamlana crocina*, *Bacillus aquimaris*, *Erythrobacter aquimaris*, and *Halomonas halophila*. The production of PHB is expected to be a good replacement for petroleum-based plastics in a number of industrial applications. A study reported by Rezk et al. [31] showed that wheat bran could be used as a substitute for starch nitrate medium for the production of PHB by *Streptomyces incanus* BK128. Danial et al. [32] isolated the *B. wiedmannii* AS-02 OK576278 strain, which was capable of producing PHB when pre-treated with various agricultural residues such as onion peels, banana peels, mango peels, orange peels, mango and rice straw were used as the carbon sources, respectively. In a similar manner, the aim of the present study was to isolate and screen PHB-producing bacteria from municipal solid waste landfill site soil samples collected from the Ha’il region of Saudi Arabia with a focus on enhancing biopolymer production, including characterization and optimization with a particular focus on the production of PHB using low-cost agricultural wastes as input material.

## 2. Materials and Methods

### 2.1. Collection of Sample and Isolation of Bacteria

During this study, different soil samples from municipal solid waste landfill sites of the Ha’il region of Saudi Arabia were collected in order to isolate PHB-producing bacteria. In 9 mL of sterilized distilled water, 1 g of soil samples were added. In a rotary shaker, samples were shaken at 120 rpm for 30 min at 37 °C. Following serial dilutions, nutrient agar plates (Hi-media^®^, Mumbai, India) were streaked with the dilutions. Incubation was conducted for 24 h at 37 °C. Purified strains were maintained at 4 °C on a nutrient agar slant and purified [33].

### 2.2. Screening the Isolates for PHB Production

A Nile blue staining technique has been implemented to screen the isolates for the presence of PHB granules. The isolated bacteria were grown in 10 mL of modified mineral salt medium (dextrose—20 g/L, peptone—2.5 g/L, yeast extract—2.5 g/L, MgSO_4_—0.2 g/L, NaCl—0.1 g/L, KH_2_PO_4_—0.5 g/L, pH—7.0) and incubated with shaking condition at 120 rpm for 48 h at 37 °C. After incubation, the cell pellet was collected via centrifugation at 6000 rpm for 10 min. The collected cell pellet was dissolved in 1 mL of sterile deionized water and then heat-fixed on glass slides. In the next step, the cells were stained with Nile blue solution (1%) (Hi-Media^®^, Mumbai, India) and viewed under a fluorescence microscope. The accumulation of PHB was observed via cells exhibiting orange granules [34]. 

### 2.3. Identification of PHB-Producing Bacterial Strain

The isolate that showed promise for PHB production was identified via 16S rRNA gene sequencing analysis. The genomic DNA from the bacterial strain was isolated using a genomic DNA isolation kit (GenEluteTM, Shanghai, China, Sigma-Aldrich^®^, Bangalore, India). By following the protocol of Sambrook et al. [35], the optical density (OD) of extracted DNA was taken at 260 and 280 nm (UV-1800, Shimadzu Spectrophotometer, Kyoto, Japan). The purity of extracted DNA was further tested by electrophoresis in agarose gel (0.8%). As part of the PCR process, universal primers 27F (5′AGAGTTTGATCMTGGCTCAG3′) and 1492R (5′CGGTTACCTTGTTACGACTT3′) were amplified in 20 µL of volume, consisting 10 ng of extracted genomic DNA, 10 pmol of each primer, 1X ReadyMixTM Taq PCR reaction mix (Sigma^®^, Bangalore, India) and nuclease-free water [36]. The reaction was carried out using a thermal cycler (Applied Biosystems Veriti^®^, Waltham, MA, USA). In the PCR cycling procedure, the following protocol was set: 95 °C for 5 min, 30 cycles of 95 °C for 1 min, 55 °C for 1 min and 72 °C for 1 min, followed by a final extension step of 72 °C for 10 min. The amplified PCR products were analyzed again on agarose gel (1%) consisting of ethidium bromide, which was visualized under the gel doc system (Bio-Rad^®^, Hercules, CA, USA). A GenEluteTM PCR clean-up kit was used to purify the PCR products, which were then sequenced by Eurofins Genomics India Pvt Ltd., Bangalore, India. In order to analyze the sequence data, the sequencing analysis software version 5.4 (Applied Biosystems^®^, Waltham, MA, USA) and the BioEdit 7.2.5 program (North Carolina State University, Raleigh, NC, USA) were used. The Basic Local Alignment Search Tool (BLAST) of NCBI was used for the analysis of obtained sequences, and the sequences were deposited to the NCBI GenBank.

### 2.4. Production and Extraction of PHB

The inoculum used for the production of PHB was prepared by transferring pure colonies of isolated bacteria in 5 mL of nutrient broth and incubating for 24 h at 37 °C. After that, 5% of the active inoculum was inoculated into 50 mL of modified mineral salt medium and further incubated at 120 rpm for 72 h at 37 °C [37]. In the following step, centrifugation at 6000 rpm for 15 min was carried out for the grown culture. Afterward, the cell pellets were collected and dried. To the dried pellet, sodium hypochlorite (10 mL) was added and incubated for 2 h at 50 °C to lyse the cells. Following that, further centrifugation at 6000 rpm for 15 min was carried out. Then, the non-PHB cell matter was removed via Whatman no. 1 filter paper. The pellet was dissolved in boiling chloroform after being washed in distilled water, acetone, and methanol. As a final step, chloroform was removed, and the PHB film was stored for future analysis [38].

### 2.5. Quantitation of PHB

The percentage accumulation of intracellular PHB was estimated based on the percentage composition of PHB in dry cell weight [39]. Cell pellets were collected via centrifugation after bacteria were grown, as described above. Cell pellets were vacuum-dried at 60 °C in order to estimate the dry weight (g/L). The accumulation of PHB (%) was determined as per the following calculation: dry weight of extracted PHB (g/L)/dry cell weight (DCW) (g/L) × 100

### 2.6. Characterization of Extracted PHB

The ultraviolet–visible (UV–Vis) spectroscopy and Fourier-transform infrared (FTIR) spectroscopy analysis were carried out to characterize the extracted PHB. The initial step was to analyze the extracted PHB using a UV–Vis spectrophotometer (Shimadzu, Kyoto, Japan), in which extracted PHB was dissolved in chloroform and analyzed in the range of 200–320 nm. The FTIR spectra were obtained between 500 to 4000 cm^−1^ (Bruker^®^, Billerica, MA, USA) [29].

### 2.7. Development of Low-Cost Production Media from Agriculture Waste

As part of the study, different agricultural waste, including rice bran, barley bran, wheat bran, orange peels and banana peels, were collected and dried for a period of five to seven days. The collected agriculture residues were grounded into a powder and used for the preparation of the extract. Then, hydrolysis of agriculture residues was carried out using (0.5–5%) sulfuric acid and autoclaving them at 121 °C for 30 min for acid pre-treatment. After the extract had been filtered, sodium hydroxide was added to the supernatant to neutralize it. A 4% concentration of hydrolysates extract, augmented with media components except for the carbon source, was used as a production medium for PHB production by the *B. flexus* HSA3 strain [32].

### 2.8. Optimization of PHB Production

As part of the optimization of the PHB production process, a number of factors were examined, using temperatures (25–45 °C), pH values (6.5–9.0), incubation periods (24–144 h), carbon sources (arabinose, maltose, sucrose, fructose, and glucose), nitrogen sources (urea, glycine, yeast extract, peptone, and yeast extract + peptone). The fermentations were conducted in 50 mL of MSM under shaking at respective conditions. Calculation of PHB production was carried out as described above. To determine the biomass, cell pellets were collected after centrifugation and dried at 60 °C and expressed as g/L [30].

### 2.9. Response Surface Methodology for Optimizing PHB Production 

Based on the conventional one-variable-at-a-time (OVAT) method, the most important independent variables (wheat bran, yeast extract, and temperature) were further statistically optimized using Box–Behnken design (BBD) via maintaining constant other variables to enhance the production of PHB. As represented in Table 1, the range and levels of the experimental variables were used. In total, 17 experiments were conducted using Design Expert^®^ (Stat-Ease, Minneapolis, MN, USA) software version 12.0 (Table 2). The BBD outcome was examined using an analysis of variance (ANOVA) and using a t-test (*p* < 0.05). The significance of each variable’s effect on PHB production was analyzed using a *t*-test (*p* < 0.05). The interaction of variables was visualized using a graphical three-dimensional (3D) surface plot [40].

### 2.10. Statistical Analysis

Three replicates of each experiment were conducted, and results are represented as means ± SDs. GraphPad Prism 5.0 (GraphPad Software, Inc., San Diego, CA, USA) was used for analysis.

## 3. Results

### 3.1. Isolation, Screening and Selection of PHB-Producing Bacteria

**Figure 1:**
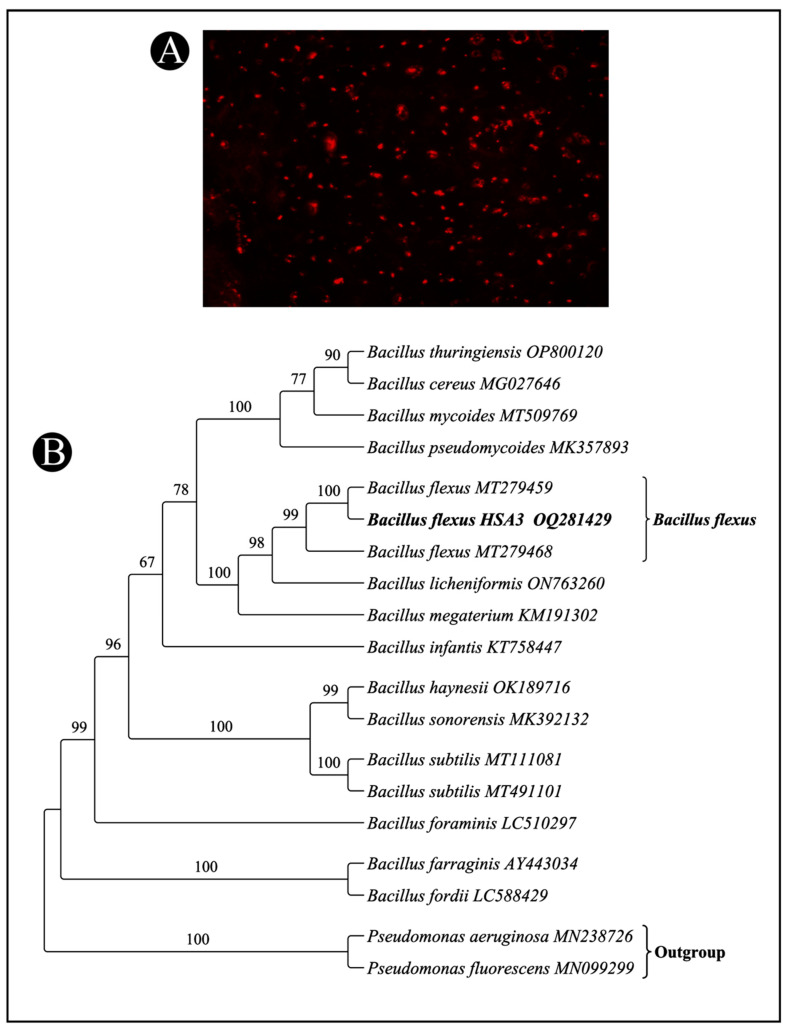
Screening of promising bacterial isolates for PHB production. (**A**) Staining of HSA3 isolates with Nile blue dye as observed under fluorescence microscope (**B**) Phylogenetic tree based on 16S rRNA nucleotide sequences of the bacterial isolates *B. flexus* strain HSA3 with other sequences of *Bacillus* strains.

From the soil samples collected in the current study, ten different bacterial isolates were obtained. The Nile blue strain was initially used to screen the isolates as a primary method of screening the bacteria for the presence of PHB. It was found that the four isolates were capable of accumulating PHB granules. Among them, one of the isolates, HSA3, was capable of producing a high amount of PHB, which was selected for further investigation (Figure 1A).

### 3.2. Identification of High-PHB-Producing Bacterial Isolate HSA3

In order to amplify the 16S rRNA gene of isolate HSA3, genomic DNA was isolated and used for PCR amplification. As a result of 16S rRNA gene amplification and sequencing, isolate HSA3 was found to share a high identity with *B. flexus*. The sequence of the gene that was obtained from isolate HSA3 has been deposited to GenBank and obtained the accession number OQ281429. Phylogenetic trees were constructed for the isolate and other *Bacillus* species (Figure 1B).

### 3.3. Characterization of PHB

**Figure 2:**
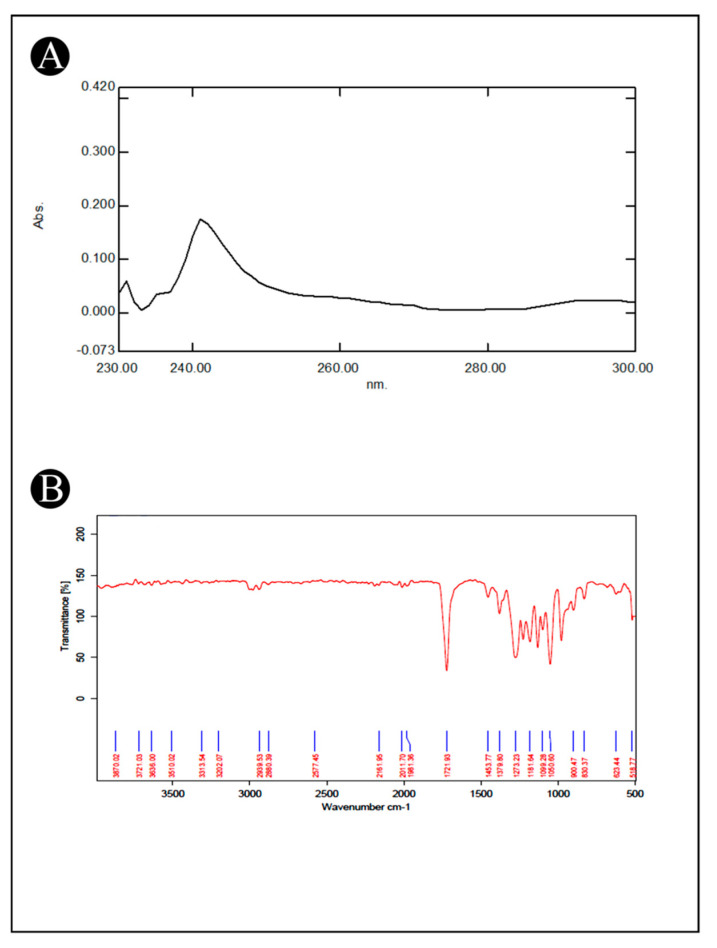
(**A**) UV–Vis spectrophotometer scanning spectrum of extracted PHB from *B. flexus* strain HSA3; (**B**) FTIR analysis of the extracted PHB from *B. flexus* strain HSA3.

The extracted polymer from the *B. flexus* HSA3 strain was examined using a UV–Vis spectrophotometer and an FTIR analysis. For the detection of PHB in the environment, UV–Vis spectroscopy has been commonly used. According to a spectroscopic analysis of the extracted PHB, the absorption spectrum of the compound was clearly symmetrical, with the peak maximum occurring at a wavelength of 241 nm (Figure 2A). FTIR spectroscopy was further used to determine various functional groups of PHB. FTIR spectra of the extracted PHB were recorded between the 4000 and 500 cm^−1^ range (Figure 2B) and displayed a sharp absorption band at 1721.93 cm^−1^, which might correspond to the carbonyl (C=O) stretching of the ester, and another band at 1273.23 cm^−1^, which might correspond to the –CH group. These bands have been labelled as a marker of PHB, extracted from various sources. As a result of stretching of the C–O bond of the ester group of the hydrocarbon, a series of bands between 1000 and 1300 cm^−1^ was observed. According to the spectra, the bands at 2939.53 and 2880.39 cm^−1^ indicate the presence of the asymmetric stretching modes of methyl (CH_3_) and methylene (CH_2_), respectively. Moreover, the bands of minor relevance observed at 3510.02 cm^−1^ are thought to be of the terminal OH group.

### 3.4. PHB Production and Incubation Time

**Figure 3:**
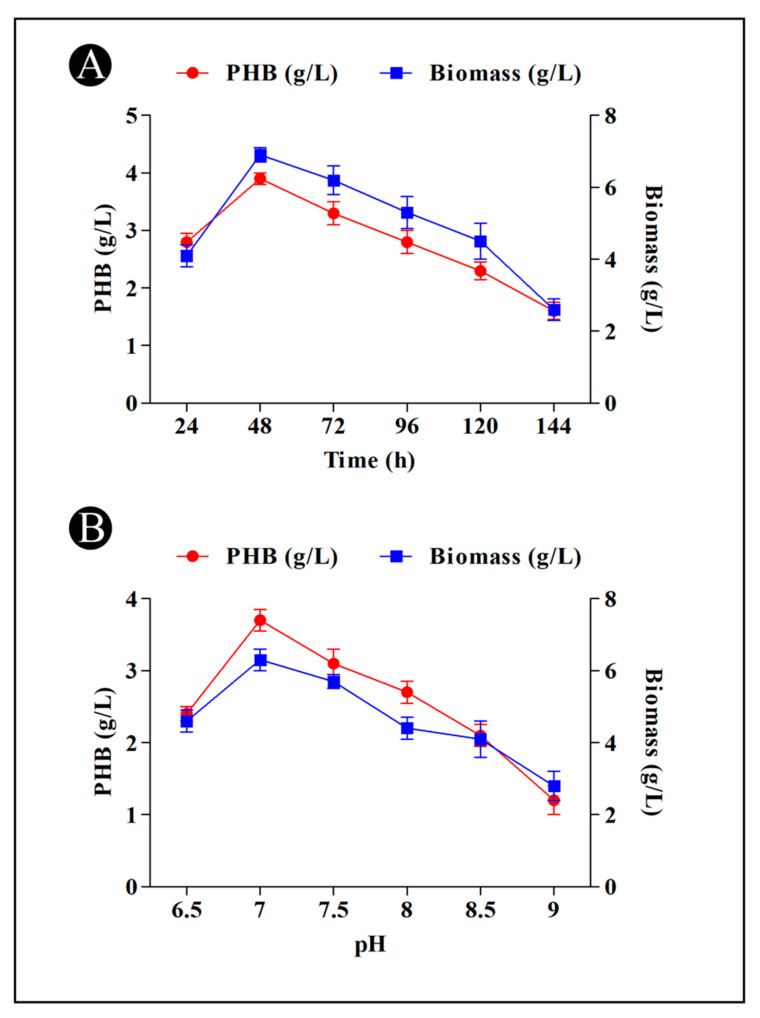
(**A**) Effect of incubation time on the PHB production by *B. flexus* strain HSA3, (**B**) Effect of pH on the PHB production by *B. flexus* strain HSA3.

The production of PHB by *B. flexus* HSA3 strain was significantly affected by incubation time. There was an increase in the content of PHB at 48 h (3.9 g/L), but after that, there was a decrease in the amount of PHB produced (Figure 3A). During the period of time when PHB production decreased, PHB was suspected to be used by the bacteria as a nutrient source, which resulted in unsuitable growing conditions due to insufficient nitrogen and carbon sources within the medium at the time.

### 3.5. PHB Production and pH

A variety of pH levels (6.5–9) were tested to determine the optimal pH level for the production of PHB. As shown in Figure 3B, the *B. flexus* HSA3 strain produced the maximum amount of PHB (3.7 g/L) at a pH of 7.0, whereas PHB production was low at pH values of 6.5 and 9.0. A neutral pH was found to be the optimal one for producing high levels of PHB.

### 3.6. PHB Production and Temperature

**Figure 4:**
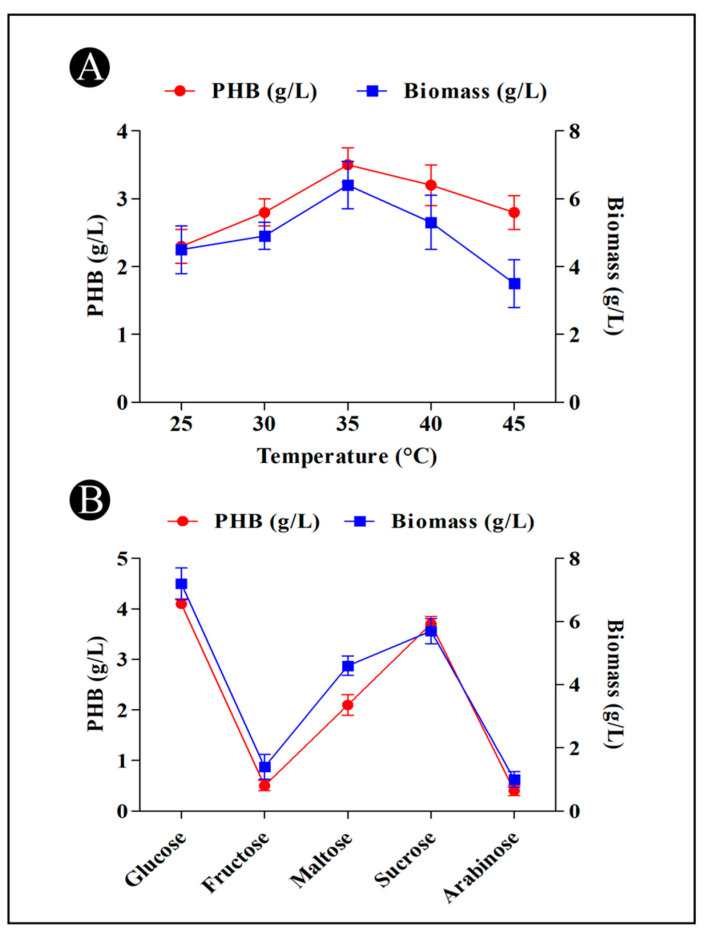
(**A**) Effect of incubation temperature on the PHB production by *B. flexus* strain HSA3. (**B**) Effect of different carbon sources on the PHB production by *B. flexus* strain HSA3.

The *B. flexus* HSA3 strain was incubated at various temperatures in production media. The maximum PHB production was found to produce at 35 °C (3.5 g/L) (Figure 4A). PHB production at different temperatures was found to vary significantly in response to the variations in temperature, which can be due to the fact that apart from optimal temperatures, other temperatures can affect the enzymes that were responsible for synthesizing PHB, resulting in a reduction in their activity.

### 3.7. PHB Production and Different Carbon Sources

PHB production in the cultures has been observed to be affected by different carbon sources (arabinose, maltose, sucrose, fructose and glucose), as represented in Figure 4B. The results of this experiment indicated that the bacteria were capable of utilizing different carbon sources in a variable manner. It has been suggested that a number of factors may have an effect on this ability, such as the type of substrates utilized and the type of enzymes that are synthesized by the bacteria. As a carbon source, glucose (4.1 g/L) was the most efficient source for producing PHB, following sucrose and maltose. A readily available carbon source such as glucose facilitates the production of PHB by bacteria.

### 3.8. PHB Production and Different Nitrogen Sources

**Figure 5:**
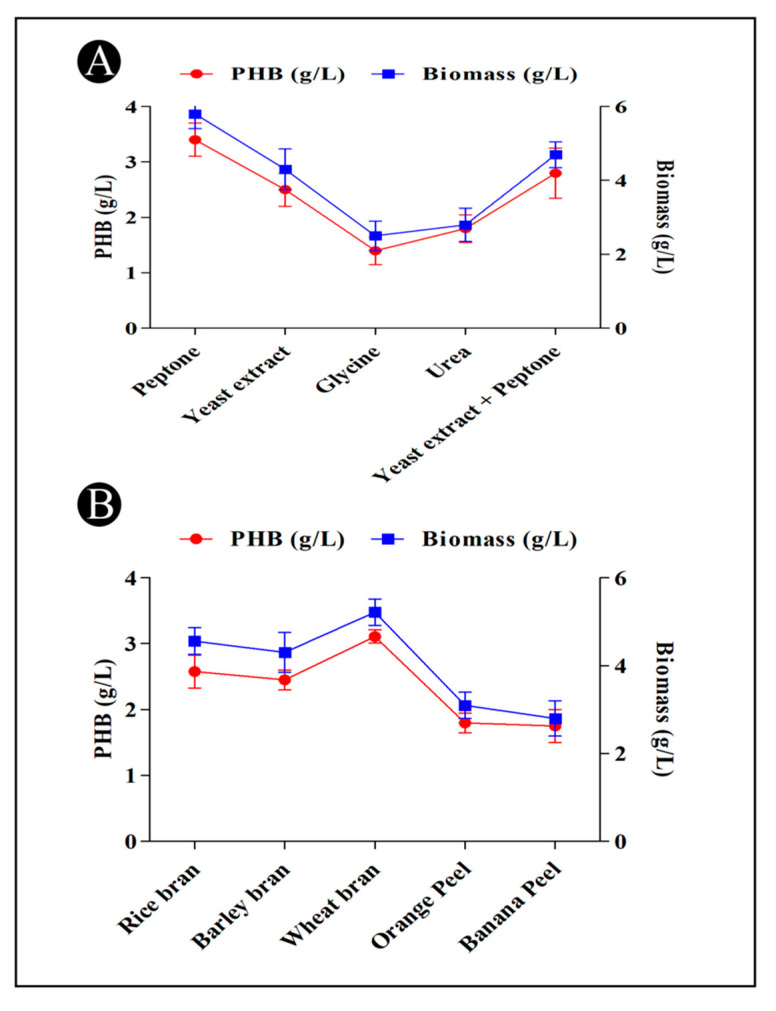
(**A**) Effect of different nitrogen sources on the PHB production by the *B. flexus* strain HSA3. (**B**) Effect of different cheap agricultural wastes on the PHB production by the *B. flexus* strain HSA3.

Different nitrogen sources (peptone, yeast extract, glycine, urea, and yeast extract + peptone) were evaluated in order to determine their effects on the production of PHB. According to the results, it became evident that *B. flexus* HSA3 held the greatest potential for producing PHB when it was grown in a medium containing peptone (3.4 g/L) (Figure 5A). The amount of PHB produced by most of the bacteria was higher when peptone was used as a source of nitrogen than the other sources. The lower nitrogen content of peptone could have been the main factor contributing to the increased PHB production observed in the experiment compared to other sources.

### 3.9. Production of PHB from Different Agricultural Wastes

Agricultural wastes are abundantly available and are a rich source of carbohydrates for use in the agriculture sector. As a result of the existence of hydrolytic enzymes in bacteria that are capable of metabolizing the complex residues of these waste carbons, many bacterial species possess the ability to utilize these varied and cheap carbon sources. The *B. flexus* HSA3 strain was able to produce PHB with a variety of agricultural waste materials (rice bran, barley bran, wheat bran, orange peel and banana peel) as its main carbon resource for the production of PHB, of which wheat bran had the highest level of production (3.11 g/L) (Figure 5B). In the present study, it was evident that the accumulation of PHB by the *B. flexus* HSA3 strain was closely related to its biomass, which consequently decreased as the biomass was reduced when the carbon was depleted, leading to the consumption of PHB by the bacteria.

### 3.10. Optimization of PHB Accumulation by Response Surface Methodology

In this study, a BBD technique was used to determine the optimal levels of the three independent variables (wheat bran, peptone, and temperature) in order to determine the PHB accumulation. The complete experimental matrix, including the actual and predicted values of responses, is presented in Table 2. The *B. flexus* HSA3 strain exhibited significant differences in terms of PHB production, indicating that the process variables within their range have an impact on PHB production (Table 2). The medium containing 20 g/L of wheat bran, 10 g/L of peptone, and a temperature of 35 °C produced the most PHB (5.45 g/L), while the medium containing 30 g/L of rice bran, 5 g/L of peptone, and a temperature of 35 °C produced the least (4.77 g/L).

As per the RSM simulation, the quadratic model was the best approach for explaining the relationship between responses and factors. The empirical relationship between the independent factors and response (PHB production) was demonstrated by a quadratic equation, as shown in the following equation:

$$\begin{array}{c} Y=+5.38-0.0363\;A+0.0503\;B+0.04\;C+0.105\;AB+0.0325\;AC\\ -0.0575\;BC-0.1835\;A^{2\;}-0.2335\;B^{2\;}-0.156\;C^{2\;} \end{array}$$

where *Y* is the predicted response (PHB production), and *A*, *B*, and *C* are the coded values of the test variables: wheat bran (g/L), peptone (g/L), and temperature (°C), respectively.

ANOVA (Analysis of Variance) is a statistical technique used to analyze the differences between two or more groups or populations. It helps to determine whether there are significant differences between the means of the groups being compared or if the differences can be attributed to random chance. A summary of ANOVA in response to the quadratic surface model for the production of PHB by *B. flexus* HSA3 strain is presented in Table 3. The values of ‘‘Prob > F” less than 0.05 indicate the significance of the model. The model F-value and *p*-value were 45.37 and <0.0001, respectively, indicating that the model was significant. The lack of fit (LOF) for this process in the present model is insignificant (0.9363), indicating that the model had acceptable terms and adequate accuracy in data prediction. The model’s accuracy and reliability were determined by evaluating predicted R^2^ and adequate precision. The “Pred R^2^” of 0.9518 is consistent with the “Adj R^2^” of 0.9615.

**Figure 6:**
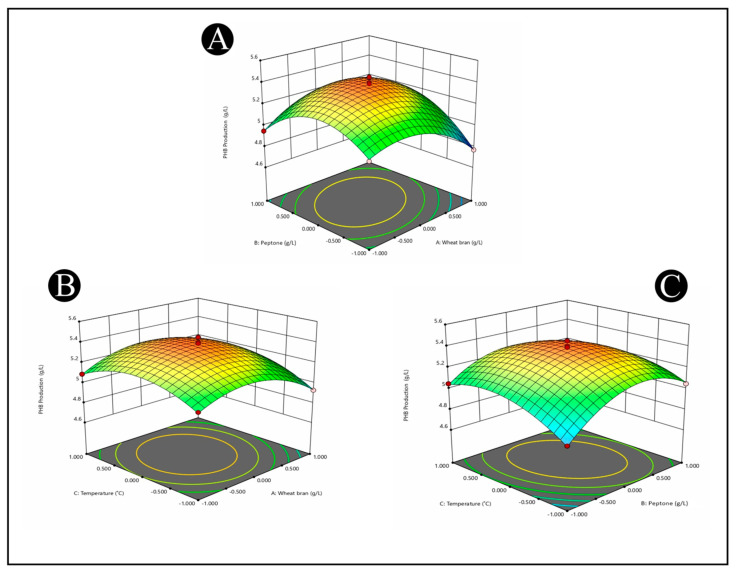
3D surface plots representing PHB production from the culture broth of *B. flexus* strain HSA3 as affected by cultural conditions of (**A**) wheat bran peptone, (**B**) wheat bran and temperature, and (**C**) peptone and temperature.

Figure 6A–C represents the profile of quadratic response surface plots for significant variable optimization (wheat bran, peptone, and temperature). The 3D response surface plot shapes describe the importance of the interaction between corresponding parameters. Each figure depicts the effect of two factors while holding the other factor constant. According to the statistics, PHB production initially increased significantly up to a certain level, and then the production was gradually reduced with its higher value. The diagnostic plots showed that the model satisfied the analysis-of-variance assumptions and also reflected the accuracy and applicability of the RSM for the optimization of the process for PHB production.

## 4. Discussion

The present study aims to identify the potential strains of bacteria that accumulate PHBs from different soil samples collected from municipal solid waste landfill sites in the Ha’il region of Saudi Arabia and then a selection of potent producers for further studies. In order to detect intracellular PHB in the isolates, a dye-based procedure was used in which orange-colored lipid granules were stained by Nile blue [41]. The high susceptibility of the lipid granules to staining with Nile blue is attributed to the high degree of incorporation of individual components of the granules into the cellular structure [42]. The results of this study indicate that the soil habitats of the Ha’il region are likely to be one of the sources of bacteria that can produce PHB. The accumulation of PHB by microbial communities in the soil is thought to be caused by low nutrient levels in the soil as a survival mechanism for bacteria in such conditions.

In order to identify bacteria accurately, 16S rRNA gene sequencing can be used as one of the methods. An essential component of the 30S ribosomal subunit of prokaryotes is the 16S rRNA subunit. This gene is encoded by a genetic marker called 16S rDNA. This modern molecular method for identifying bacteria has been proven to be reliable in recent times and replaced the traditionally used method, which was based on the phenotypic characteristics of bacteria [43]. After the 16S rRNA sequencing method, the isolated HSA3 strain was identified as *B. flexus* strain HSA3, which was the most active isolate accumulating PHB granules among other isolates. In many previous studies, different species of *Bacillus* have been reported as ideal producers of PHB [44,45]. 

When UV–Vis scans were performed on PHB extracted from the present study, peaks were observed in the range of 230–240 nm. PHB is evident in this range of peaks [46]. The FTIR analysis results showed strong stretching H bonds created by the terminal OH groups at 3510 cm^−1^. Other studies have also reported similar results [43,44]. The peaks at 2939 and 2880 cm^−1^, respectively, correspond to methyl and methylene C–H stretching groups. There are other studies that also showed similar results, including the ones by Kumalaningsih et al. [47] (2925.81 cm^−1^) and Anish et al. [39] (2932 cm^−1^). Specifically, Randriamahefa et al. [48] hypothesized that the absorption bands of 1721 cm^−1^ were PHA markers and corresponded to carbonyl C=O stretches of the ester group distributed within the chain of exceedingly ordered crystalline structures. In comparison, these peaks were similar to the standard peaks for scl-PHA and mcl-PHA [47]. In PHA, short chain lengths (scl-PHA) have 3–5 carbon atoms, whereas medium chains (mcl-PHA) have 6–14 carbon atoms [49]. The polymer extracted from the *B. flexus* strain HSA3 showed prominent absorption bands, confirming it was poly-β-hydroxybutyrate.

Upon 48 h of incubation, *B. flexus* strain HSA3 achieved a maximum rate of growth and accumulated the most PHB. As a result, the biomass and the PHB production of a particular strain were associated with growth conditions and the PHB production of a particular strain is correlated with its biomass. As the biomass increases, the bacteria begin to accumulate PHB to the maximum level, and the accumulation of PHB decreases as the bacteria reaches the peak biomass production level. There is a possibility that the bacteria are forced to use the accumulated PHB as a source of energy due to nutrient depletion [50]. As a result of glucose as a carbon source, the *B. flexus* strain HSA3 yielded the highest content of PHB. It has been observed that Adwitiya et al. [51] reported similar results when using glucose as a source of carbon in *R. sphberoides* N20 and Gomez et al. [52] when using glucose in *Alcaligenes latus*. The carbon source glucose is readily assimilated by bacteria and therefore encourages them to produce more PHB [53,54]. The growth and production of PHB were also demonstrated by a number of other bacteria, including *Vibrio azureus* BTKB33, *Bacillus megaterium*, *Alcaligens eutrophus*, and other *Bacillus* sp., which also showed that it is a highly effective carbon source for growing and producing PHB [55]. A temperature of 37 °C was found to be the optimal temperature for the growth of *B. flexus* strain HSA3 and accumulation of PHB. The PHB as well as biomass yields increased substantially until 37 °C but started to decline sharply as temperature increased. It was also reported by Noha et al. [56] that maximum cell density and accumulation of PHB were achieved at 37 °C after 48 h of culture. Changing temperature can alter the content of PHB because extreme temperatures slow down the metabolism (enzyme activity) of microorganisms, resulting in a reduction in their ability to produce PHB. The PHB is produced at the highest rates by *B. flexus* strain HSA3 when the pH is at 7.0. These results are consistent with those of Hong et al. [57], Sharma and Harish [58], and Singh et al. [59]. Moreover, peptone as a nitrogen source was found to produce the highest percentage of PHB per dry cell weight, followed by yeast extract. Due to the relatively low nitrogen content in peptone, this could be attributed to the fact that the C/N ratio of those molecules was increased, which in turn favored more PHB accumulation [60]. In terms of cell dry weight and PHB content, glycine was found to have the lowest value. In bacterial media, nitrogen concentrations have a large effect on PHB production, as reported by Paramjeet et al. [61].

The major obstacle in the commercialization of bioplastics is the high cost of their production. As a strategy for the cost-effective production of PHB, the use of renewable sources, which presents a low-cost carbon feedstock, and the development of bacterial strains with the ability to produce high quantities of intracellular PHB utilizing these low-cost substrates are required [32,62,63,64]. In the agricultural sector, such waste is abundantly available and is a rich source of carbohydrates that can be used as a renewable resource [30,65]. Considering the fact that these wastes have little economic value, most of them are used as cattle feed. *Bacillus* species possess innate abilities to utilize cheap and diverse carbon waste materials due to the fact that they possess hydrolytic enzymes which allow them to decompose these complex residues. In this way, native *Bacillus* strains are currently being explored in an industrial setting for the production of PHB using agro-wastes as a source of carbon [62,66,67].

A number of studies have demonstrated that different strains of bacteria utilize a variety of carbon sources. *Ralstonia eutropha* was reported to utilize glycerol as a carbon source by Taidi et al. (1994) [68]. The PHB production was not affected by the carbon source used in the culture, either sucrose or glycerol. As a carbon source for microorganisms, fatty acids can be supplemented from fermented fruit and vegetable residues [69]. In fed-batch mode, *Azotobacter chroococcum* produced 25 g/L of PHB when soluble starch was used for carbon [70]. A study of PHB production by 11 different *Bacillus* species was conducted by Chen et al. (1991) [71]. In this study, it was found that a maximum of 50% of the dry cell weight of bacteria was composed of PHB (*w/v*). According to a study conducted on 29 *Bacillus* strains for the production of PHB, *B. megaterium* was found to have the highest production rate of 0.207 g/L and the highest productivity percentage of 48.13%. The lowest level of PHB was found in *B. subtilis* K1 at 6.53% [72].

It is important to note that the present study has reported higher production of PHB (3.11 g/L) compared to the recent studies of Van-Thuoc et al. (2007) [73] and Ramadas et al. (2009) [54], which used wheat bran as the carbon source and reported 1.08 g/L and 1.06 g/L PHB concentrations, respectively. Protein concentration is high in wheat bran hydrolysate [73]. A number of agro-industrial residues, such as potato starch, babassu, soy cake [74], cane molasses, and whey [75], have been reported to be useful in the production of PHB. As reported by Fukui and Doi (1998) [76], some plant oils, such as olive oil, corn oil, and palm oil, were good carbon substrates for the production of PHB by *R. eutropha*. Coconut oil was found to be one of the best carbon sources for *Comamonas testosteroni* by Thakor et al. (2005) [77]. Rusendi and John (1995) [78] reported to have produced 77% of the dry biomass weight from waste potato starch hydrolysate in the production of PHB using waste potato starch hydrolysate.

Investigations regarding the production of bioplastic from date syrup as one of the most abundant and inexpensive by-products in Saudi Arabia have been conducted in several studies [79,80]. In the previous studies, high PHB production (3.6 g/L) and biomass (5.1 g/L) of *Bacillus* sp. were recorded using 5% date syrup [81], while maximum PHB production (5 mg/mL) by *Bacillus* sp. was observed with 8% date syrup [79]. Moreover, PHB production by *B. megaterium* using date syrup and beet molasses was high, reaching 50–52% PHB [80]. In addition, the high PHB production by *Pseudomonas* sp. (15–20 g/L) obtained using rice bran, dates, and soy molasses corresponded to a PHB content of 90.9%, 82.6%, and 91.6%, respectively [82]. Other renewable resources were also used for PHB production, such as waste paper hydrolysate by *Burkholderia sacchari* [83], affording the highest PHB production (1.6 g/L) and PHB content (44.2%), with a PHB yield of 15% and productivity of 0.033 g/L/h. Additionally, kitchen waste-derived organic acids were used as a carbon source for PHB production by *C. necator*, affording a PHB content of 52.79% with a PHB yield and productivity of 0.38 g/g and 0.065 g/L/h, respectively; the productivity increased in the fed-batch culture to 0.242 g/L/h [84].

A range of environmental variables, such as carbon and nitrogen sources, as well as factors such as incubation time, temperature, and pH in the initial stage of fermentation, all play an important role in bacterial fermentation [85] as these are all known to influence the production of PHB by other microorganisms [32,86]. A flask-scale RSM using BBD was performed in order to enhance the production/accumulation of PHB. RSM-optimized medium yielded the highest yield of 5.45 g/L PHB production when compared to the original unoptimized medium. In comparison to the unoptimized medium, ~1.33 fold more PHB was produced. There is evidence to suggest that the RSM is an effective tool for enhancing the production of PHB by a wide variety of microorganisms, as has been previously described. There has been recent evidence that RSM can be used in order for higher production of PHB from glucose in a newly engineered strain of *C. necator* NSDG-GG [87]. *Methylobacterium* sp. was successfully used by the RSM to increase PHB production via methanol alone as the carbon source [88]. RSM was also found to improve the production of PHB by the *B. drentensis* strain BP17 when pineapple peel was used as the sole carbon source [89]. Moreover, a study conducted by Hassan et al. [90] also indicated that the *B. subtilis* strain grown in rice bran was capable of producing PHB in an optimized manner through RSM using Box–Behnken design technique with efficiency. Thus, RSM results of the present study for an increase in PHB production from a low-cost agro-waste source can be potentially applied for application and commercialization. However, the plastic nature and biodegradability of the extracted PHB need to confirm before being applied in real applications via different tests, including dynamic immersion tests [91]. A dynamic degradation test is a way to measure the rate at which a bioplastic degrades under real-world conditions. This type of test is important because it provides insight into how a bioplastic will behave in different environments and over time, which is critical information for manufacturers and consumers. To perform a dynamic degradation test for bioplastics, a sample of the material is placed in a natural environment, such as soil or water, where it is exposed to a range of environmental conditions, such as temperature, humidity, and microbial activity. The sample is then periodically removed and analyzed for changes in weight, mechanical properties, and molecular structure. The goal of a dynamic degradation test is to simulate real-world conditions as closely as possible in order to gain insight into how a bioplastic will behave in the environment. This information can be used to optimize the bioplastic’s composition and manufacturing process, as well as inform consumers about the expected lifespan and environmental impact of the product [92].

Overall, PHB production from agricultural waste can offer several economic benefits over the production of petrochemical-based plastics. The production of PHB from agricultural waste can be cheaper than petrochemical-based plastic production, as the raw materials (i.e., agricultural waste) are often readily available and inexpensive compared to petrochemical-based plastics. PHB production from agricultural waste can reduce dependence on fossil fuels, which are becoming increasingly scarce and expensive. This can also reduce the environmental impact of plastic production by decreasing the number of greenhouse gases produced [24,26]. The production of PHB from agricultural waste material can have economic benefits as it can create a value-added product from a waste stream. One of the economic benefits of producing PHB from agricultural waste material is that it can provide a new source of revenue for farmers and agri businesses. Instead of discarding agricultural waste material, it can be converted into a valuable product, which can be sold to various industries that use biodegradable polymers. Moreover, the production of PHB from agricultural waste material can also contribute to reducing the environmental impact of waste disposal. By converting agricultural waste material into a biodegradable polymer, it can reduce the amount of waste that ends up in landfills or incinerators, which can result in cost savings associated with waste disposal [27].

The production of PHB from agricultural wastes is an area of active research, and while there are many potential benefits to this approach, there are also several limitations to be considered. Agricultural wastes are a diverse group of materials with varying compositions, which can make it challenging to achieve consistent and reproducible results in PHB production [62]. This can also make it difficult to scale up the process for industrial production. Agricultural wastes may contain contaminants such as heavy metals or pesticides, which could be harmful to the microorganisms used in the PHB production process. Careful screening and treatment of the feedstock may be necessary to minimize these risks. Moreover, the yield of PHB production from agricultural wastes may be lower than from other sources, which can limit the economic viability of the process. The technology used in PHB production from agricultural wastes is still developing, and there may be technical limitations that need to be overcome to make the process more efficient and cost-effective [66]. Overall, while the production of PHB from agricultural wastes is a promising area of research, it is important to carefully consider these limitations and work towards developing solutions to overcome them in order to achieve the full potential of this approach [67]. However, the production of PHB from agricultural waste material may have some economic challenges. The process of producing PHB requires specialized equipment and technology, which can result in high capital and operating costs. Additionally, the market for biodegradable polymers may not be as developed as traditional polymers, which can result in lower demand and market prices for biodegradable polymers.

## 5. Conclusions

This study examined the production of PHB by ten different bacterial strains isolated from the soil samples collected from the municipal solid waste landfill sites of Ha’il region, Saudi Arabia, indicating the significance of soil habitats of the Ha’il region. A high level of PHB production by the isolate *B. flexus* strain HSA3 was obtained after 48 h at pH 7.0, 35 °C, with glucose as carbon and peptone as nitrogen sources. The extracted polymer was characterized by UV–Visible spectrophotometer and FTIR analysis to confirm its structure as PHB. The B. flexus strain HSA3 was capable of efficiently utilizing a variety of cheap agricultural wastes, including rice bran, barley bran, wheat bran, orange peel and banana peel, which led to the production of PHB, with wheat bran being the most effective carbon source. RSM optimization through BBD for PHB synthesis was highly effective at increasing polymer yields. Compared to an unoptimized medium, this strain can increase PHB production by approximately 1.3-fold under optimum conditions when obtained from the RSM. Therefore, the RSM can be a powerful tool for optimizing PHB production. Using the RSM through the BBD, a high degree of efficiency was demonstrated in augmenting the polymer yield through the optimization of PHB synthesis. Based on the results of this study, it can be concluded that the *B. flexus* strain HSA3, which is a viable alternative to petroleum-based plastics in a variety of useful industrial applications, produces PHB under optimal conditions. However, further research is required to achieve economically viable commercial production in fermenters, as well as to optimize the process and develop cost-effective downstream processing methods. Additionally, the properties of PHB produced from agricultural waste need to be characterized, and the process needs to be scaled up to meet the demand for sustainable plastics.

## Figures and Tables

**Table 1:** Variables optimized by BBD for PHB production.

Name of Variable with Code	Unit	Range and Levels		
		−1	0	+1
Wheat bran (A)	g/L	10	20	30
Peptone (B)	g/L	5	10	15
Temperature (C)	°C	30	35	40

**Table 2:** BBD, along with experimental and predicted values of the dependent variable for PHB production.

Run	A	B	C	PHB Production (g/L) ± SD	
				Experimental	Predicted
1	0	1	1	5.02	5.03
2	−1	0	−1	5.08	5.07
3	0	0	0	5.32	5.38
4	0	0	0	5.35	5.38
5	0	0	0	5.39	5.38
6	1	−1	0	4.77	4.77
7	−1	−1	0	5.04	5.05
8	0	0	0	5.45	5.38
9	0	−1	1	5.05	5.04
10	1	0	−1	4.93	4.93
11	−1	0	1	5.09	5.09
12	0	−1	−1	4.85	4.84
13	1	0	1	5.07	5.08
14	0	1	−1	5.05	5.06
15	−1	1	0	4.95	4.95
16	1	1	0	5.1	5.08
17	0	0	0	5.4	5.38

**Table 3:** ANOVA for PHB production as a function of independent variables.

Source	Sum of Squares	*df*	Mean Square	*F*-Value	*p-*Value Probe > *F*
Model	0.6333	9	0.0704	45.37	<0.0001 *
A-Wheat bran	0.0105	1	0.0105	6.78	0.0352 *
B-Peptone	0.021	1	0.021	13.55	0.0078 *
C-Temperature	0.0128	1	0.0128	8.25	0.0239 *
AB	0.0441	1	0.0441	28.44	0.0011 *
AC	0.0042	1	0.0042	2.72	0.1428
BC	0.0132	1	0.0132	8.53	0.0223 *
A²	0.1418	1	0.1418	91.43	<0.0001 *
B²	0.2296	1	0.2296	148.04	<0.0001 *
C²	0.1025	1	0.1025	66.08	<0.0001 *
Residual	0.0109	7	0.0016		
Lack of Fit	0.001	3	0.0003	0.1316	0.9363 ^ns^
Pure Error	0.0099	4	0.0025		
Cor Total	0.6441	16			

*Df—*degree of freedom; ns—not significant; *—significant.

## Data Availability

All data generated or analyzed during this study are included in this article.
